# Supplementary figures and images for: Spatial and temporal dynamics of leptospirosis in South Brazil: A forecasting and nonlinear regression analysis
Source: PLoS Negl Trop Dis. 2023 Apr 14;17(4):e0011239. doi: 10.1371/journal.pntd.0011239 (PMC10132658; doi:10.1371/journal.pntd.0011239)

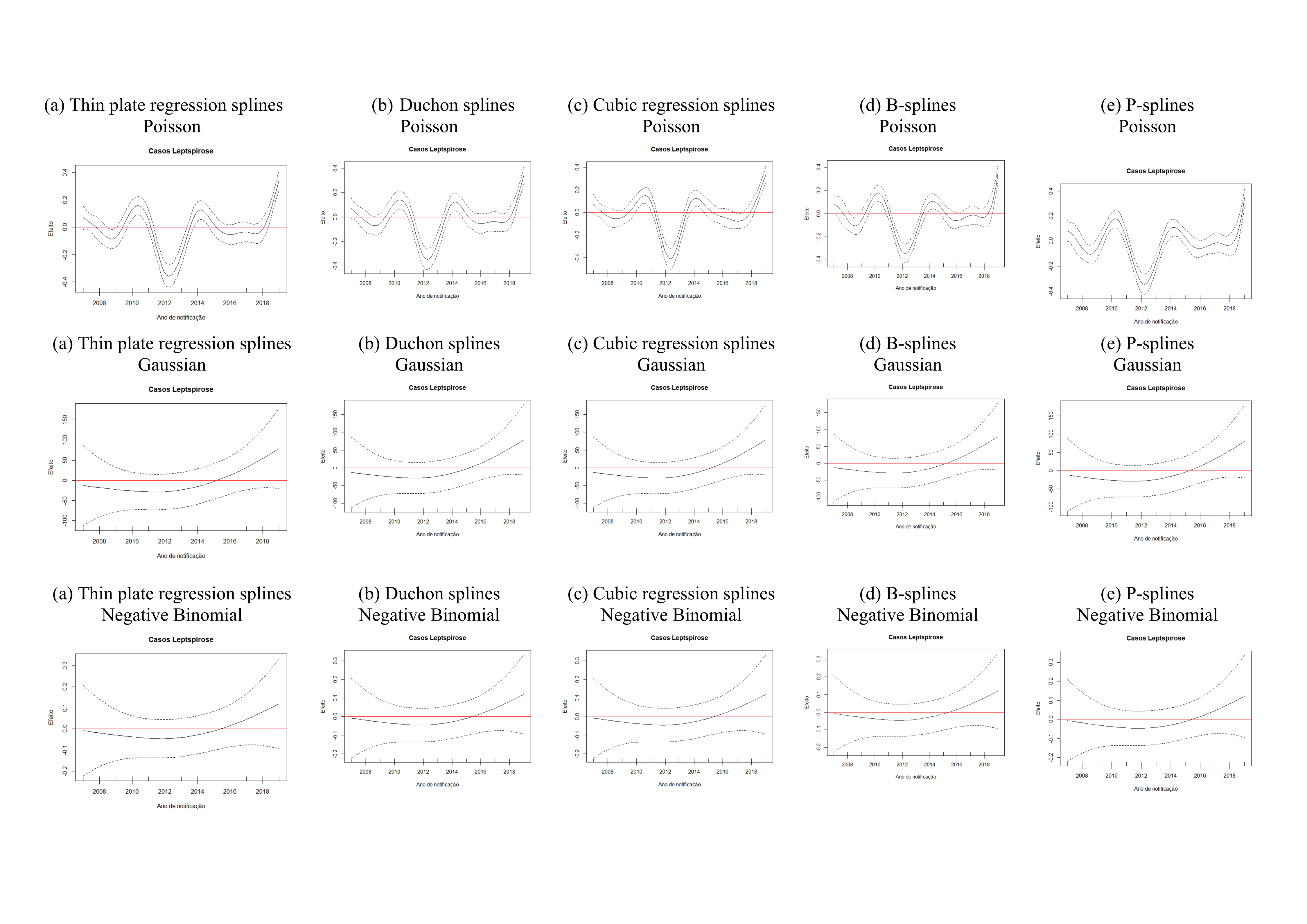

Supplement: S1 Fig — Goodness-of-fit graphics of possible Generalized Additive Models(GAM) for Poisson, Gaussian and Negative Binomial models using: (a) thin plate regression; (b) duchon splines; (c) cubic regression spline; (d) b-spline; and (e) p-splines smoothness terms. (TIF) [file pntd.0011239.s001.tif]
