# Supplementary material for: Spatial and temporal dynamics of leptospirosis in South Brazil: A forecasting and nonlinear regression analysis
Source: PLoS Negl Trop Dis. 2023 Apr 14;17(4):e0011239. doi: 10.1371/journal.pntd.0011239 (PMC10132658; doi:10.1371/journal.pntd.0011239)
Supplement: S1 Table — p<0,001; 1Un-Biased Risk Estimator (UBRE), 2Generalized Cross Validation and 3Restricted Maximum Likelihood were used to identify appropriate smoothness. (PDF) [file pntd.0011239.s002.pdf]

SI 1 Table: Goodness-of-fit summary of possible GAM models using different Families and Smoothness terms

Family	Smooth classes	Thin plate regression splines	Duchon splines	Cubic regression splines	B-splines	P-splines
Poisson	Intercept	-10.07***	-10.07***	-10.07***	-10.07***	-10.07***
	Year	8.80***	10.04***	8.82***	8.64***	8.67***
	Deviance explained	75.2%	75.1%	75.9%	74.8%	74.8%
	UBRE[1]	4.56	4.77	4.44	4.60	4.60
Gaussian	Intercept	457.01***	457.01***	457.01***	457.01***	457.01***
	Year	1.66	1.66	1.66	1.66	1.65
	Deviance explained	24.7%	24.7%	24.7%	24.7%	24.8%
	GCV[2]	9560.72	9562.73	9559.32	9556.60	9531.81
Negative binomial	Intercept	-10.06***	-10.01***	-10.06***	-10.06***	-10.06***
	Year	1.56	1.55	1.56	1.56	1.556
	Deviance explained	16.2%	16.2%	16.2%	16.3%	16.3%
	REML[3]	80.44	81.76	79.27	79.01	79.01

***p<0,001; [1] Un-Biased Risk Estimator (UBRE), [2]Generalized Cross Validation and [3]Restricted Maximum Likelihood were used to identify appropriate smoothness.
