# Supplementary material for: Spatial and temporal dynamics of leptospirosis in South Brazil: A forecasting and nonlinear regression analysis
Source: PLoS Negl Trop Dis. 2023 Apr 14;17(4):e0011239. doi: 10.1371/journal.pntd.0011239 (PMC10132658; doi:10.1371/journal.pntd.0011239)
Supplement: S2 Table — Applied model; 1AIC: Akaike’s Information Criterion; 2Mean absolute percentage error. (PDF) [file pntd.0011239.s003.pdf]

SI 2 Table: Goodness-of-fit summary of possible Seasonal Autoregressive Integrated Moving Average (SARIMA) models

Model	Leptospirosis				
	AIC[1]	Schwarz	Hannan-Quin	MAPE[2]	U-Theil
***(1,2,3) x (1,0,1)$_{12}$**	**119,3199**	**98,03**	**110,65**	**50,09**	**0,68**
(1,1,1) x (1,1,1)$_{12}$	198,5531	107,95	116,74	118,36	0,71
(2,1,1) x (1,1,1)$_{12}$	198,5176	215,37	204,81	124,15	0,79
(3,1,1) x (2,1,1)$_{12}$	199,6267	222,31	208,24	115,63	0,76
(1,2,3) x (2,1,2)$_{12}$	214,0748	222,63	238,42	92,91	0,69
(2,2,3) x (1,1,1)$_{12}$	210,8367	234,49	220,45	118,19	0,71
(1,2,2) x (1,0,1)$_{12}$	137,7296	136,88	186,06	62,05	0,68

*Applied model; [1]AIC: Akaike's Information Criterion; [2]Mean absolute percentage error
