# Supplementary material for: Spatial and temporal dynamics of leptospirosis in South Brazil: A forecasting and nonlinear regression analysis
Source: PLoS Negl Trop Dis. 2023 Apr 14;17(4):e0011239. doi: 10.1371/journal.pntd.0011239 (PMC10132658; doi:10.1371/journal.pntd.0011239)
Supplement: Attachment — Submitted filename: Comments on Manuscript PNTD-D-22-01154.pdf [file pntd.0011239.s004.pdf]

Comments for Manuscript PNTD-D-22-01154

1 Summary

Manuscript title: Spatial and temporal dynamics of leptospirosis in South Brazil: a forecasting and nonlinear regression analyses

Main research question/contribution: The manuscript focuses on the implementation of Generalized Additive Models (GAM) and Seasonal Autoregressive Integrated Moving Average (SARIMA) models to forecast leptospirosis incidence cases and to identify regions (clusters) with high leptospirosis incidence and lethality rates in the state of Rio Grande do Sul.

Overall impression: While the authors do an acceptable job on explaining concepts regarding the statistical models being implemented, the background information provided on these models are well documented in the literature. Authors should provide only the necessary information that is relevant to their work, include references for further details, and spent more time explaining how the best model was chosen (add more details about the steps taken). In the manuscript, it is shown that meaningful and important analysis results are derived by analyzing the leptospirosis data due to its public health concern. However, the manuscript requires several modifications and reconstruction. The references need major modifications, since they do not follow journal guidelines.

Reviewer decision: In its present form, the manuscript does not seem suitable for publication. Due to the importance of leptospirosis in South Brazil and the issue presented because of underreporting of cases, this analysis serves a basis for further studies and analysis. Therefore, I encourage the authors to resubmit a revised manuscript, by providing a point-by-point response on how the comments below were addressed.

2 Specific comments

2.1 Major issues

- Authors should improve explanations about the implementation of the models. Include additional results (as a supplementary material or within the manuscript) that supports the selection process of the model
- It is not sufficient to say that "*we followed Baquero et al. (2018) and used a Poisson likelihood and cubic splines with 5 knots on all predictors*". The authors need to explain the rationale of why this Poisson-GAM is adopted for the presented work
  - The authors mentioned that the models were trained, but no further discussion is provided
  - Negative binomial and Gaussian likelihoods were also used in the training process, but no comments are provided about these comparisons

- While the authors provide plots of the results, they omitted vital information on how the models were ultimately chosen
  - No predictors were explicitly mentioned
  - Results on predictive performances and model comparison (AIC) need more explanation (see first bullet provided)
- Explanations in Methods and Results sections need to be improved/restructured
- When explaining the prediction component, the intervals provided should be presented as prediction intervals, not as confidence intervals
- In Figure 4, use prediction bands instead of lines, and use different color (not light green)
- I suggest the authors to get editing help from someone with full professional proficiency in English
- The references of the manuscript should follow the journals guidelines. It is somewhat difficult to read through the manuscript when several citations are located within a sentence given the style provided
  PLOS Guideline: "References are listed at the end of the manuscript and numbered in the order that they appear in the text. In the text, cite the reference number in square brackets (e.g., "We used the techniques developed by our colleagues [19] to analyze the data"). PLOS uses the numbered citation (citation-sequence) method and first six authors, et al."

2.2 Minor issues

- Abbreviations in the Abstract should be avoided
- In the sentence "*Leptospirosis behavior over the years was evaluated by time series analyses, using generalized additive models (GAM) and SARIMA models to predict its future incidence.*" in the Abstract, remove the two abbreviations. Provide the full names of the methods in the Abstract
- Provide the full model name for SARIMA when first introduced in the text
  Example: Seasonal Autoregressive Integrated Moving Average (SARIMA)
- Check several words not in English (Portuguese): endêmic, áreas
- Change keyword from "surviellence" to "surveillance"
- Remove sentence "*Leptospirosis is a persistent health issue in Brazilian south*" from Abstract since the first sentence states a similar idea
- Provide meaning for "sp." as specie(s) and then use Leptospira sp.
- Provide an Author Summary section, in which the research findings are accessible to a wide audience. See journal guidelines for this component
- Abbreviations are being defined in multiple occasions. Define them once (only the first time they are mentioned)
- Do not start a new paragraph with "Thus,..."
- There are several typos within the text
  Example of typos: colected, clursters, redictor

- Remove “(clursters)” in the sentence “*This analysis was performed to detect spatial clusters with a higher concentration of high incidences (clursters) of leptospirosis*”

- Final versions of the Figures should be in high quality resolution (use TIFF). It is not possible to Zoom-in very well in the figures

- Revise sentence “*GAM models provide a framework for generalizing a general linear model, allowing the variables nonlinear functions additivity*”. The phrase “generalizing a general linear model” is not providing the correct idea

- Improve explanation in second paragraph of subsection Generalized additive models (GAM). For instance: what are $g(\mu_i)$, Y_i, x_i, and the index i, within the explanation? This paragraph is an example of a text that can be either removed from the text or improved

- Provide citation of the R packages: “mgcv” and “tidyverse”

- In “*(60 deaths, LR 8.9%)*”, please provide the meaning of LR. Is it lethality rate? Please, define the abbreviation before using the abbreviation

- When referencing a figure or table, use expression “Figure 1” or “Table 1”

- What happened with the data from 2007 to 2009 in the incidence and lethality maps, and heatmaps? It is not clear from the text

- Do not use the phrase “*GAM models*”. The M in GAM means model

- Provide references for all softwares. I do not see references for R and Gretl softwares

- The following paragraph should be included as an Ethic Statement: “*The study was approved by the Ethics Committee of the Faculdade de Medicina of Universidade Federal de Pelotas, CAAE 46714421.0.0000.5317, in accordance with all ethical principles and current legislation for research involving human beings. In this way, data confidentiality was guaranteed and used only for research purposes.*’

- In Discussion section, address what are possible future works (e.g. further analysis into under-reporting and missclasification)
