# Supplementary material for: Spatial and temporal dynamics of leptospirosis in South Brazil: A forecasting and nonlinear regression analysis
Source: PLoS Negl Trop Dis. 2023 Apr 14;17(4):e0011239. doi: 10.1371/journal.pntd.0011239 (PMC10132658; doi:10.1371/journal.pntd.0011239)
Supplement: Attachment — Submitted filename: PNTD-D-22-01154_reviewed.pdf [file pntd.0011239.s005.pdf]

PLOS Neglected Tropical Diseases

Spatial and temporal dynamics of leptospirosis in South Brazil: a forecasting and non-linear regression analyses

--Manuscript Draft--

**Manuscript Number:**	PNTD-D-22-01154
**Full Title:**	Spatial and temporal dynamics of leptospirosis in South Brazil: a forecasting and non-linear regression analyses
**Short Title:**	leptospirosis in South Brazil: a forecasting and non-linear regression analyses
**Article Type:**	Research Article
**Keywords:**	Keywords: Leptospira interrogans; Time series; Spatial analyses; surviellence.
**Abstract:**	Despite leptospirose being endêmic in most Brazilian regions, the south region is responsible for the highest morbidity and mortality outcomes within the country. The objective of this study is analyse the spatial and temporal dynamics of leptospirosis cases to identify the temporal trends and high-risk areas for transmission, as well as to propose a model to predict the incidence of the disease in the South of Brazil. An ecological study of leptospirosis cases in the 497 municipalities of the state of Rio Grande do Sul, Brazil, was carried out during the period of 2007 and 2019. The spatial distribution of disease incidence in southern Rio Grande do Sul municipalities was evaluated, while higher incidence of the disease were identified using hotspot density technique. Leptospirosis behavior over the years was evaluated by time series analyses, using generalized additive models (GAM) and SARIMA models to predict its future incidence. The highest incidence was observed in the central and the metropolitan region of Porto Alegre, which was also identified as a cluster with high incidence and high risk of contagion. In the analysis of the incidence temporal series, peaks were identified in the years 2011, 2014 and 2019. The SARIMA model predicted a fall in incidence in the first half, followed by an increase in the second half of 2020. Leptospirosis incidence prediction proved to be adequate and can be used as a tool for health services. Leptospirosis is a persistent health issue in Brazilian south. Temporal and spatial clustering of cases highlight the demand for intersectoral surveillance and control public policies, with a focus on reducing inequalities among different municipalities in Brazil.
**Additional Information:**	
**Question**	**Response**
**Financial Disclosure**<br><br>Enter a financial disclosure statement that describes the sources of funding for the work included in this submission. Review the submission guidelines for detailed requirements. View published research articles from *PLOS NTDs* for specific examples.<br><br>This statement is required for submission and **will appear in the published article** if the submission is accepted. Please make sure it is accurate.	This study received specific funding from the Research Support Foundation of the State of Rio Grande do Sul, intended only for equipment such as notebooks and the like, from the Fundaçao de Amparo a Pesquisa do Estado do Rio Grande do Sul Rio Grande do Sul.<br>There were no grants awarded.<br>There were no awards attribution.<br>https://sig.fapergs.rs.gov.br/index.php<br><br>Funders had no role in study design, data collection and analysis, publication decision, or manuscript preparation.

Powered by Editorial Manager® and ProduXion Manager® from Aries Systems Corporation

**Unfunded studies**<br><br>Enter: *The author(s) received no specific funding for this work.*<br><br>**Funded studies**<br><br>Enter a statement with the following details:<br>• Initials of the authors who received each award<br>• Grant numbers awarded to each author<br>• The full name of each funder<br>• URL of each funder website<br>• Did the sponsors or funders play any role in the study design, data collection and analysis, decision to publish, or preparation of the manuscript?<br>• **NO** - Include this sentence at the end of your statement: *The funders had no role in study design, data collection and analysis, decision to publish, or preparation of the manuscript.*<br>• **YES** - Specify the role(s) played.<br><br>* typeset	
**Competing Interests**<br><br>Use the instructions below to enter a competing interest statement for this submission. On behalf of all authors, disclose any competing interests that could be perceived to bias this work—acknowledging all financial support and any other relevant financial or non-financial competing interests.<br><br>This statement **will appear in the published article** if the submission is accepted. Please make sure it is accurate. View published research articles from *PLOS NTDs* for specific examples.	The authors have declared that no competing interests exist.

Powered by Editorial Manager® and ProduXion Manager® from Aries Systems Corporation

**NO authors have competing interests**<br><br>Enter: *The authors have declared that no competing interests exist.*<br><br>**Authors with competing interests**<br><br>Enter competing interest details beginning with this statement:<br><br>*I have read the journal's policy and the authors of this manuscript have the following competing interests: [insert competing interests here]*<br><br>* typeset<br><br>This statement is required for submission and **will appear in the published article** if the submission is accepted. Please make sure it is accurate and that any funding sources listed in your Funding Information later in the submission form are also declared in your Financial Disclosure statement.	
**Data Availability**<br><br>Authors are required to make all data underlying the findings described fully available, without restriction, and from the time of publication. PLOS allows rare exceptions to address legal and ethical concerns. See the PLOS Data Policy and FAQ for detailed information.	Yes - all data are fully available without restriction

A Data Availability Statement describing where the data can be found is required at submission. Your answers to this question constitute the Data Availability Statement and **will be published in the article**, if accepted.<br><br>**Important:** Stating 'data available on request from the author' is not sufficient. If your data are only available upon request, select 'No' for the first question and explain your exceptional situation in the text box.<br><br>Do the authors confirm that all data underlying the findings described in their manuscript are fully available without restriction?	
**Describe where the data may be found in full sentences. If you are copying our sample text, replace any instances of XXX with the appropriate details.**<br><br>• If the data are **held or will be held in a public repository**, include URLs, accession numbers or DOIs. If this information will only be available after acceptance, indicate this by ticking the box below. For example: *All XXX files are available from the XXX database (accession number(s) XXX, XXX.*).<br>• If the data are all contained **within the manuscript and/or Supporting Information files**, enter the following: *All relevant data are within the manuscript and its Supporting Information files.*<br>• If neither of these applies but you are able to provide **details of access elsewhere**, with or without limitations, please do so. For example:<br><br>*Data cannot be shared publicly because*	All relevant data are within the manuscript and its Supporting Information files.

Powered by Editorial Manager® and ProduXion Manager® from Aries Systems Corporation

of [XXX]. Data are available from the XXX Institutional Data Access / Ethics Committee (contact via XXX) for researchers who meet the criteria for access to confidential data.<br><br>*The data underlying the results presented in the study are available from (include the name of the third party and contact information or URL).*<br>• This text is appropriate if the data are owned by a third party and authors do not have permission to share the data.<br><br>* typeset	
Additional data availability information:	Tick here if the URLs/accession numbers/DOIs will be available only after acceptance of the manuscript for publication so that we can ensure their inclusion before publication.

Manuscript
Click here to access/download;Manuscript;Spatial and temporal dynamics of leptospirosis SUL.docx

Spatial and temporal dynamics of leptospirosis in South Brazil: a forecasting and non-linear regression analyses

TELES, Alessandra Jacomelli[1]; BOHM, Bianca Conrad[2]; SILVA, Suellen Caroline Matos[2], BRUHN, Nádia Campos Pereira[3]; BRUHN, Fábio Raphael Pascoti[4].

[1]Secretaria Municipal de Saúde de Herval, Rio Grande do Sul, Brasil.

[2]Programa de Pós Graduação em Veterinária da Universidade Federal de Pelotas, Capão do Leão, Rio Grande do Sul, Brasil.

[3]Universidade Federal de Pelotas, Centro de Integração do Mercosul, Pelotas, Rio Grande do Sul, Brasil.

[4]Universidade Federal de Pelotas, Departamento de Veterinária Preventiva, Centro de Controle de Zoonoses, Capão do Leão, Rio Grande do Sul, Brasil.

*suellen.carol.as@gmail.com

Abstract

Despite leptospirose being endêmic in most Brazilian regions, the south region is responsible for the highest morbidity and mortality outcomes within the country. The objective of this study is analyse the spatial and temporal dynamics of leptospirosis cases to identify the temporal trends and high-risk areas for transmission, as well as to propose a model to predict the incidence of the disease in the South ~~of~~ Brazil. An ecological study of leptospirosis cases in the 497 municipalities of the state of Rio Grande do Sul, Brazil, was carried out during the period of 2007 and 2019. The spatial distribution of disease incidence in southern Rio Grande do Sul municipalities was evaluated, while higher incidence of the disease were identified using hotspot density technique. Leptospirosis behavior over the years was evaluated by time series analyses, using generalized additive models (GAM) and SARIMA models to predict its future incidence. The highest incidence was observed in the central and the metropolitan region of Porto Alegre, which was also identified as a cluster with high incidence and high risk of contagion. In the analysis of the incidence temporal series, peaks were identified in the years 2011, 2014 and 2019. The SARIMA model predicted a fall in incidence in the first half, followed by an increase in the second half of 2020. Leptospirosis incidence prediction proved to be adequate and can be used as a tool for

health services. Leptospirosis is a persistent health issue in Brazilian south. Temporal and spatial clustering of cases highlight the demand for intersectoral surveillance and control public policies, with a focus on reducing inequalities among different municipalities in Brazil.

Keywords: Leptospira interrogans; Time series; Spatial analyses; surviellence.

Introduction

The febrile illness leptospirosis is one of the most widespread emerging zoonoses worldwide (Adler and Moctezuma, 2010). It has a high transmission capacity for individuals in social and environmental vulnerability conditions (Clazer et al., 2015; Gonçalves et al., 2016; Buffon, 2018). Therefore, in Brazil, it constitutes a relevant public health problem, with social, health and economic impact, high hospital costs, lost working days and high lethality (Brasil, 2019; Martins and Spink, 2020). The etiological agent of the infection is Leptospira sp., which has the ability to live in varied environments for prolonged periods. Pathogenic species affect humans and wild, domestic and synanthropic animals, which can become carriers and contribute to the microorganism spread in nature (Benacer et al., 2016).

In Brazil, leptospirosis is an endemic disease and is present in all regions of the country. It has a high incidence, with an annual average of 3,926 confirmed cases and a death rate of 8.9%, between 2007 and 2016, with the highest number of cases registered in the Southeast and South regions (Brasil, 2018; Marteli et al. ., 2020; Galan et al., 2021).

Leptospirosis is easily overlooked and relatively little is known about it, as few studies are carried out about the disease, and that’s why it is considered a neglected disease. In this sense, reliable data on its incidence and prevalence in different areas are still scarce (World Health Organization, 2003). Thus, we consider that the analysis of real data on the incidence of confirmed leptospirosis cases collected from information systems derived from the official surveillance of the disease in Brazil can provide contributions to the elucidation of the difficulties in control and to the dynamic understanding of the occurrence of this endemic disease in the country. Observations can be used as tools to help health managers to design prevention and promotion public health actions (Martinez and Silva, 2011; Souza, Uberti and Tassinari, 2020).

Transmissible diseases surveillance data is useful to managers while monitoring daily incidence trends, and the usefulness of this data can be extended with the help of statistical analysis. It can be used to find out gaps in the notification system, to identify the disease incidence peak periods in a season. In addition to those benefits, predictive analytics gives us scope to predict the future burden of the disease as well as to early predict an epidemic (Promprou, Jaroensutasinee and Jaroensutasinee, 2006; Lal et al., 2012; Ho and Ting,02015; Kapagunta and Chetty, 2021).

Thus, the objective of this paper was to analyze the spatial and temporal distribution of leptospirosis incidence rate, in the period between 2007 and 2019, in Rio Grande do Sul, Brazil and to predict the expected incidence of the disease in a subsequent period. Based on the results we expect to contribute to the development of more effective strategies for leptospirosis control in a region of high incidence, such as southern Brazil.

Methods

A ecological study was carried out based on data colected in leptospirosis surveillance routine in the state of Rio Grande do Sul, between 2007 and 2019. The dynamics of the disease in the state was analyzed through spatial and temporal statistical analyses. An incidence prediction model for subsequent years was built.

Study location

Rio Grande do Sul is the southernmost state in Brazil, divided into 497 municipalities. It has an estimated population of 11,422,973 inhabitants in 2020 (IBGE, 2010), an area of 281,730.149 km^2, with a population density of 39.79 inhabitants/km^2. It is located at latitude 27°03"42"" and 33°45"09"" South, and 49°42"41"" and longitude 57°40"57"" West. The climate is subtropical temperate, with great seasonal variation in temperature, with average temperatures ranging between 15 and 18°C, and different rainfall volume among regions, with average annual precipitation of 1,500 mm (Socioeconomic Atlas of Rio Grande do Sul, 2020).

The literacy rate is above 95%. The Gross Domestic Product *per capita*, according to statistical data from 2017, is close to R$37 thousand/year. In 2015, 93.7% of the population had directly or indirectly garbage collection; 40% had sewage collection network around their homes (IBGE, 2010).

There are three major economic regions: 1) in the south with greater concentration of land, large livestock farms and mechanized planting of rice, soybeans and wheat. This area also has greater income inequality; 2) northeast region, which includes the state capital, with more industries and predominantly small properties; and 3) the northern region, mostly colonized by European immigrants, with greater forest cover, valleys and plains with small agricultural lands (Atlas Socioeconômico do Rio Grande do Sul, 2020)

Data collect

Since 2000, notification of human leptospirosis cases is mandatory through the Ministry of Health's Information System for Notifiable Diseases (acronym in Portuguese SINAN), through a passive surveillance system. In 2006, the notification form was updated to include minor modifications and it became effective across the country in 2007 (Galan et al., 2021retirar de 3). Information on leptospirosis confirmed cases, reported in the *Sistema de Informação de Agravos de Notificação* (SINAN), in Rio Grande do Sul, covering the period from 2007 to 2019, was obtained through Datasus system, Tabnet, from *Ministério da Saúde* (Brazil, 2016; Brazil, 2019). The cases included in this study were confirmed by clinical-epidemiological or laboratory criteria in accordance with the Epidemiological Surveillance Guide of the Ministry of Health (IBGE, 2021).

Statistical analysis

An ecological study of time series and spatial analysis was developed, which make it possible to identify geographical temporal patterns of the disease in Rio Grande do Sul. Using leptospirosis data over 13 years of observation, incidence rates (per 100,000 inhabitants) and lethality (%) were calculated using the population projection of Instituto Brasileiro de Geografia e Estatística, for each year of interest (IBGE, 2021).

Those indicators were annually mapped - in all municipalities in Rio Grande do Sul - in order to verify the existence of clusters and, therefore, analyze the disease risk in different regions of the state. In addition, incidence rates were analyzed using temporal regression models and forecasting, using SARIMA modeling.

Spatial clusters

Hotspot analysis evaluates events distribution in a given area. It is possible to visually identify the concentration of the event and indicate concentration areas where the phenomenon frequently occurs. This analysis was performed to detect spatial clusters with a higher concentration of high incidences (clursters) of leptospirosis, called hotspots, between 2010 and 2019, using the Getis-OrdGi statistic (Anselin, 1995).

The hotspots are observed as central points, where there is a greater intensity of the analyzed event in the study area, and considering each central point circular distance, it performs a score of all points within the sample influence radius, with a event smoothing (Marteli et al., 2020; Chaiblich et al., 2017). Thus to visualize the Hotspot analysis with the highest leptospirosis incidence áreas, 'Heat Maps' were constructed (Qgis, 2021).

Spatial statistical analyzes were performed using the Geographic Information System of Quantum GIS software (QGIS), version 3.4.7.

Generalized additive models (GAM)

Generalized additive models (GAM) represent an extension of the generalized linear model, as proposed by Hastie and Tibshirani (1987) and Hastie and Tibshirani (1990). Generalized additive models represent an alternative for modeling nonlinear relationships that do not have a defined shape (Conceição et al., 2001). They are based on non-parametric functions, called smoothing curves, in which the association shape is defined by the data (Hastie and Tibshirani; 1990; Conceição et al., 2001). GAM models provide a framework for generalizing a general linear model, allowing the variables nonlinear functions additivity (Ravindra et al., 2019).

With this replacement, it is not necessary to assume a linear relationship between $g(\mu_i)$ and the explanatory variables, nor is it necessary to previously know the relationship form, but it is possible to estimate it from a data set (Conceição et al., 2001). This estimated function, also called a smoothed curve, is nothing more than some kind of Y_i average values in the vicinity of a given x_i value, which allows describing the shape, and even revealing possible nonlinearities in the studied relationships, since it does not have the rigid structure of a parametric function (Conceição et al., 2001).

In order to select the best GAM, we followed Baquero et al. (2018) and used a Poisson likelihood and cubic splines with 5 knots on all predictors. We proceeded with

a Poisson likelihood (we trained equivalent models with negative binomial and Gaussian likelihoods), to tune the type of spline (shrinkage cubic or cubic) and the upper limit on the degrees of freedom (df) associated with the spline (k = df-1 = {3,4,5,6,7,8}) (Baquero et al., 2018).

The general formula of GAM with Poisson likelihood was:

$$y_i \sim Poisson\ (Z_i)$$
$$\log(Z_i) = b_0 + \sum s_j\ (x_{ij}k),$$

where y_i was the observation i, Z_i is the linear predictor for the observation i, b_0 is the intercept, s_j is the spline for predictor x_{ij} and k is the number of knots (Baquero et al., 2018).

GAM models allow a wide range of distributions for the adopted response variable, as well as linkage functions to measure the redictor variables effects on the dependent regressors, as reported by McCullagh and Nelder (1984) and Hastie and Tibshirani (1990). Thus, in this study, GAMs were adjusted in order to verify the association form lepotospirosis occurrence of over time. Gross rates in Rio Grande do Sul were adjusted. The response variables were the observed reported and confirmed cases with Poisson distribution. The models linear predictor was formed by the notification year variable (2007 -2019) with a smoothing function (spline) wih the offset term being the population natural logarithm exposed in each notification year. The analyzes were performed using R software and “mgcv” and “tidyverse” packages.

SARIMA modelling

Time series analysis was used to describe leptospirosis behavior between 2007 and 2019 and to forecast the disease incidence in 2020. Trend elements evaluation, seasonality, cyclical variation, association and random variation are time series analyses components. That assessment makes it possible to understand the implicit processes over time and, thus, providing important information for illness public health policies planning (Antunes, 2015).

In the present study, for time series analysis, trend and seasonality components were evaluated, as well as the Seasonally Automatic Regressive Integrated Moving Average (SARIMA) model adjustment.

Time series can be decomposed into the equation:

$$Y_t = T_t + S_t + a_t$$

Where trend (T_t) refers to the increase or decrease on observations over a period; seasonality (S_t) shows fluctuations occurring in periods shorter than one year; while the random component or error (a_t) shows irregular random oscillations (Morettin and Toloi, 2006). When evaluating leptospirosis incidence through a monthly series, it is possible to find several random factors, such as climate and health policies changes, among others. Those factors are known as white noise, as they are random elements that occur due to chance and are not related to cyclical variations or trend.

SARIMA time series model was used to assess trend and seasonality components and their adjustment (Morettin and Toloi, 2006). Cases from 2007 to 2019 were used to build the logical system. Data from the year 2019 was used for validation and to predict the last year of the study, 2020. In order to validate the adopted model choice, the real values were compared with the predicted ones and their 95% confidence interval was calculated (95% CI). In addition, Mean Absolute Percent Error (MAPE) values and Theil-U statistic were used. Values lower than 1 (one) indicated forecast adequacy.

Monthly incidences were transformed by adding the first difference to the logarithmic variable to make stationary series. In the months in which leptospirosis occurrence was 0 (zero), one unit was assigned. The least squares method was used to verify trends and seasonality ($p<0.05$). The augmented Dickey-Fuller test (ADF) was performed to test data stationarity ($p<0.05$) (Dickey and Fuller, 1979).

Each model adequacy was verified using the residuals Autocorrelation Function (ACF) and Partial Autocorrelation Function (PACF) histogram graphs, in addition to the Ljung-Box test to investigate those residuals randomness (Ljung and Box, 1978). In order to compare different models adjustment, the Akaike Information Criterion (AIC) and Hannan-Quinn and Schwarz alternative models were used. Time series analyzes were performed using Gretl 1.9.12 software (GNU Regression, Econometrics and Time-series Library).

The study was approved by the Ethics Committee of the Faculdade de Medicina of Universidade Federal de Pelotas, CAAE 46714421.0.0000.5317, in accordance with all ethical principles and current legislation for research involving human beings. In this way, data confidentiality was guaranteed and used only for research purposes.

Results

From 2010 to 2019, 4,760 cases and 238 deaths from human leptospirosis were confirmed in Rio Grande do Sul. The average incidence rate was 4.06 cases per 100,000 inhabitants and lethality rate of 5%. Leptospirosis' incidence and lethality rates spatial distribution are shown in figure 1. In several regions of the state, high leptospirosis incidence rates were observed, with emphasis in the Metropolitana, Vales and Norte Macroregion. In addition, it was observed that 220 municipalities did not confirm any case in the entire period, while deaths from leptospirosis were recorded in 59 municipalities, with the highest number of deaths recorded in the capital, Porto Alegre (60 deaths, LR 8.9%).

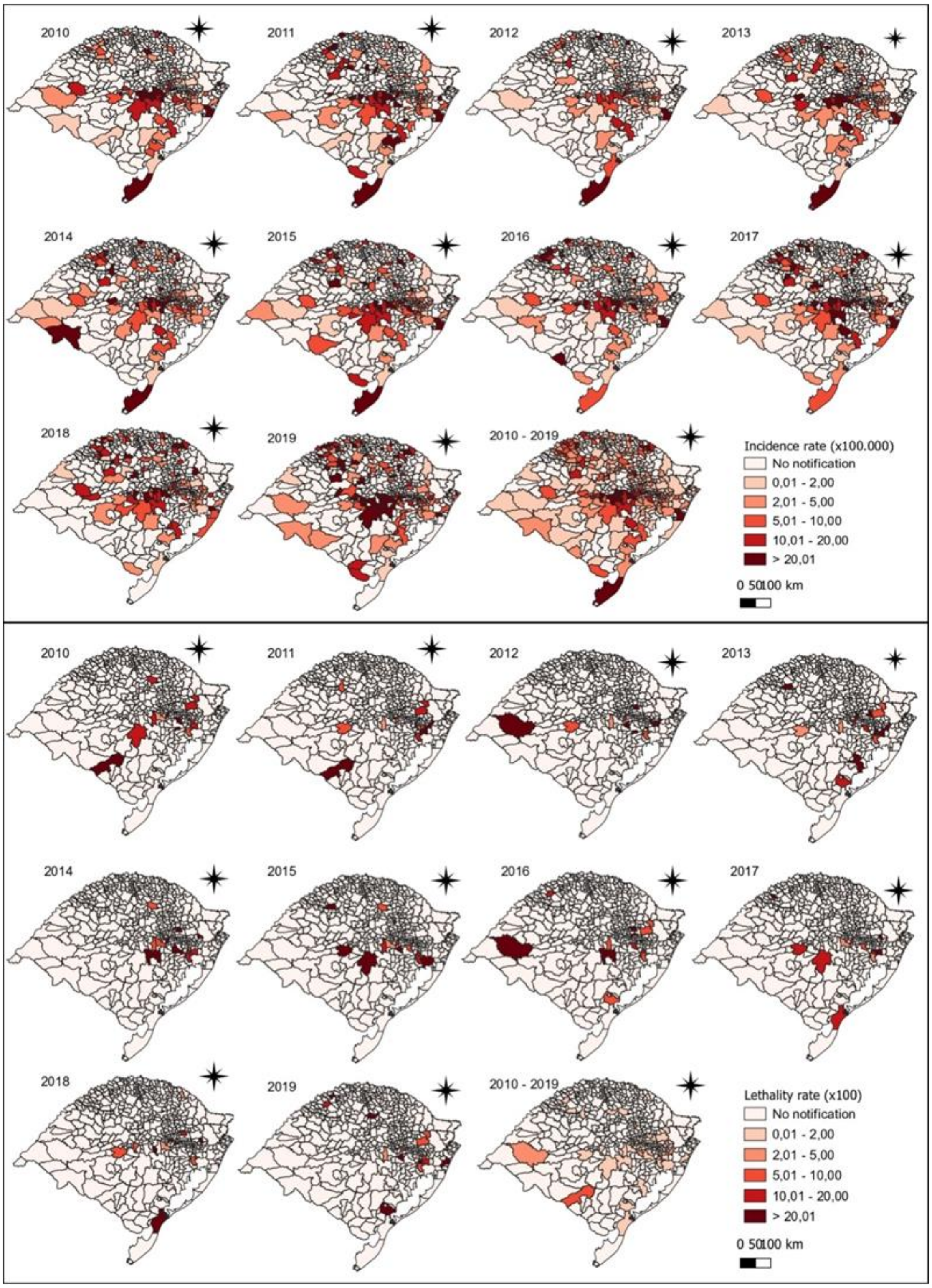

Figure 1. Leptospirosis incidence and lethality rate spatial distribution in Rio Grande do Sul municipalities, Brazil, 2010 to 2019 (incidence rate: cases/ 100,000 inhab.; lethality rate: %).

Figure 2 presents annual maps of the areas with the highest concentration of high leptospirosis incidence, represented with density levels according to shade. The hotspots, central points represented in red color, demonstrate that there is a high concentration of high leptospirosis incidence rates, around it is observed that the tonality is softening (yellow color) with the decrease in the incidences of the disease. The mapping shows, in all years, hotspots in the metropolitan macroregion of Porto Alegre, that is, a concentration of high incidence rates. Possibly in these places there is a high risk of leptospirosis transmission. The municipalities in this area have high population density and smaller territorial extension.

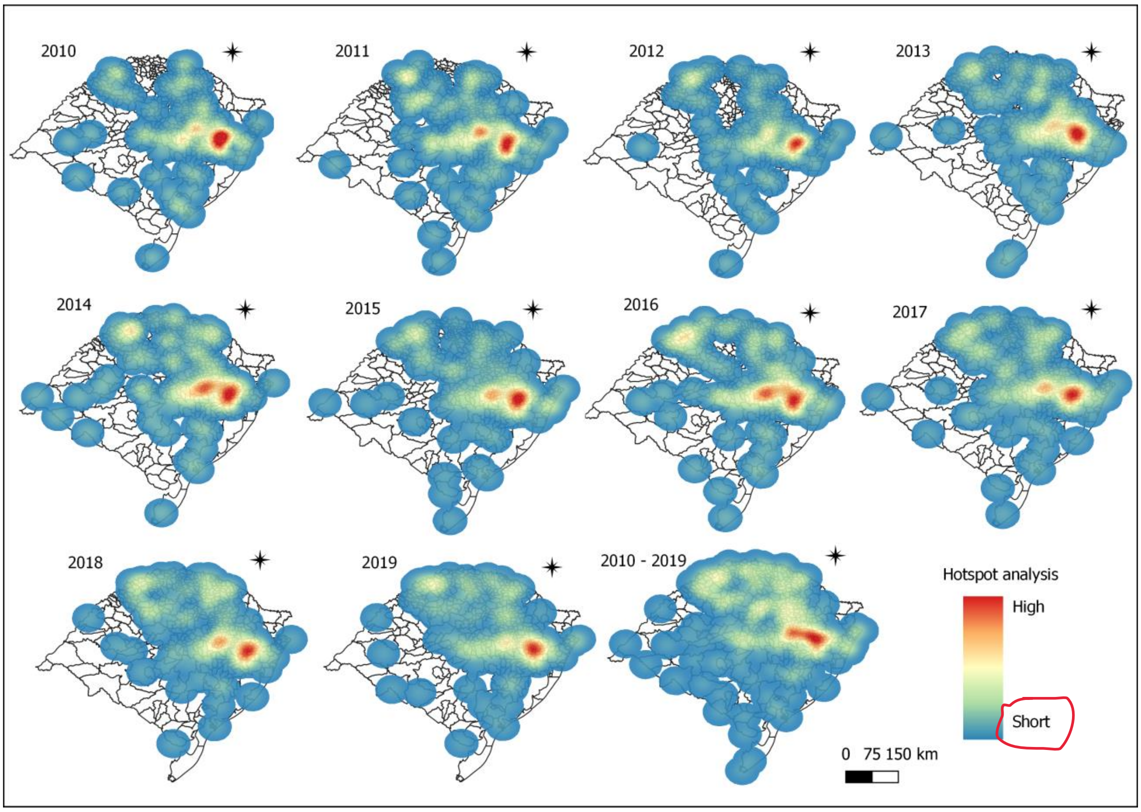

Figure 2. Hotspot analysis of Leptospirosis incidence, from 2010 to 2019, in Rio Grande do Sul, Brazil.

Using GAM, a leptospirosis non-linear regression model for the cases over the years (Figure 3A), it was possible to observe an increase in the disease occurrence risk in Rio Grande do Sul from 2010, with a peak in 2011, followed by a decrease in risk until 2012. Between 2013 and 2019, the disease risk annually increased, with new peaks in the years 2014 and especially 2019. Similarly, in the analyzed leptospirosis incidence time series between 2007 and 2019, it was observed incidence peaks in the years 2011

and 2019. In addition, after performing the logarithmic transformation, it was possible to identify the seasonality of the disease in the months of January, February, March and April (P<0.05) (Figure 3B). After differentiating the time series, the necessary stationarity for the SARIMA modeling was confirmed using ADF test, in addition to the ACF and PACF (Figure 3B).

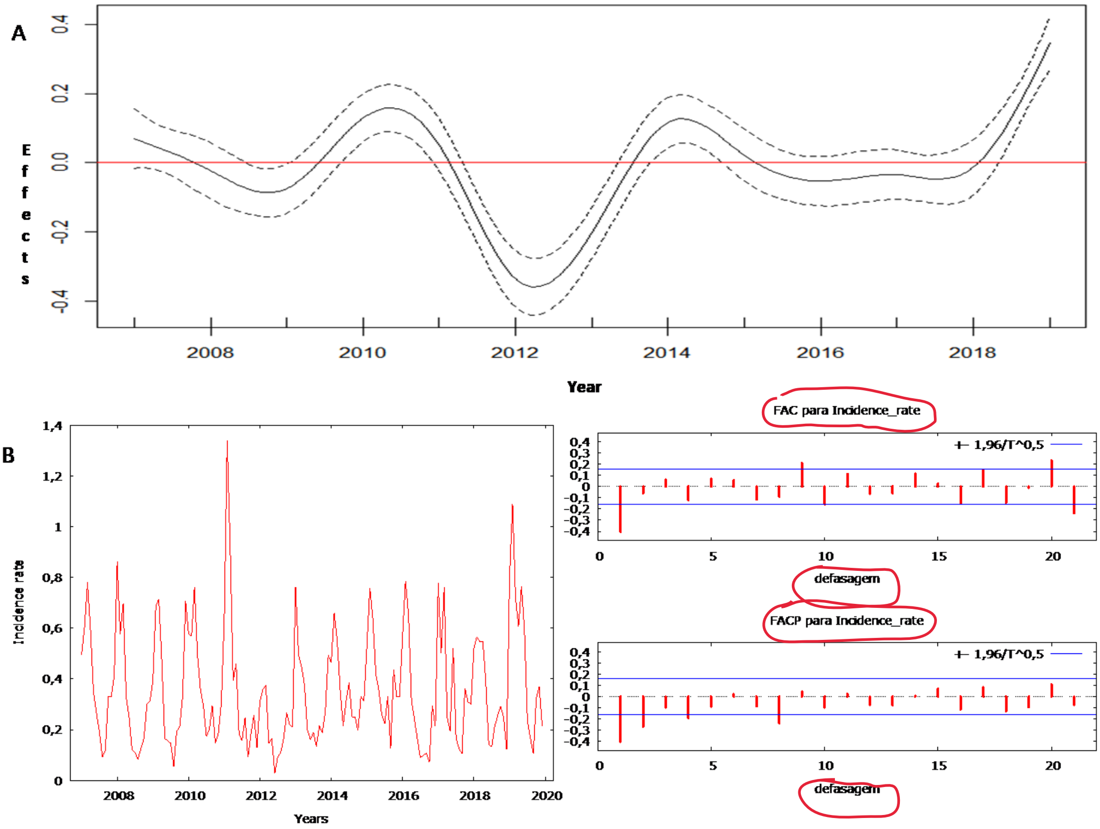

Figure 3. Leptospirosis incidence temporal distribution between 2007 and 2019 in Rio Grande do Sul, Brazil, estimated by Generalized Additive Models (GAM) with 95% confidence interval (95% CI) (p<0.01, R^2= 75.2%) (A); and time series descriptive evaluation in the period, with the Autocorrelation Functions (ACF) and the Partial Autocorrelation Function (PACF) correlograms (B).

In leptospirosis incidence time series modeling, the best model found was SARIMA (1,2,1) (1,0,1). When verifying the model adequacy, through Theil-U statistic, the value of 0.683 and Mean Absolute Percentage Error (MAPE) of 50.0% were obtained, indicating acceptable forecast errors, suggesting an adequate proposed model to predict leptospirosis future incidence. After adjusting the model, the original series data (red line) and the predicted values (blue line) in the period under analysis were

compared, which allowed leptospirosis incidence prediction in Rio Grande do Sul, for the year of 2020, and the confidence interval (green line) (figure 4). The model considered the 12-month seasonal pattern and predicted a drop in incidence in 2020 (Figure 4).

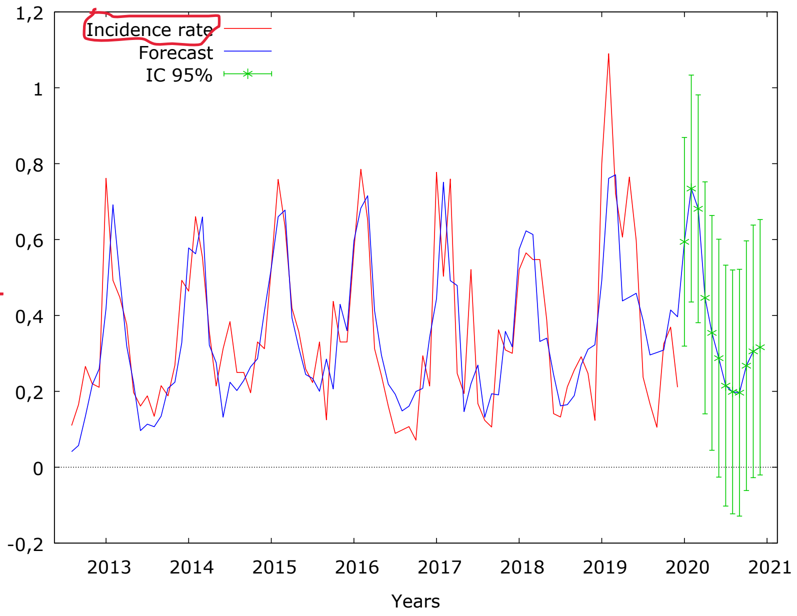

Figure 4. Monthly leptospirosis incidence time series distribution in Rio Grande do Sul, using data from 2007 to 2019, and predicted values for 2020, with confidence interval, after adjustment.

Discussion

Our study demonstrated the complexity of the endemic occurrence of leptospirosis in a large and heterogeneous Brazilian state as Rio Grande do Sul. The last years of the studied time-series highlighted the difficulties in controlling this disease, given the increase in the number of cases of leptospirosis. In Brazil, leptospirosis is a neglected disease and epidemiological data may be non-existent and inaccurate (WHO, 2013). The often nonspecific or silent clinical presentation contributes to its underreporting. Still, there is a lot of difficulty in raising awareness about the need of notification of suspected disease by health professionals (Martins and Spink, 2020; Souza et al., 2010).

In this context, it was observed that the incidence rates distribution in Rio Grande do Sul varied greatly in the different regions of the state, and in 20 municipalities it was very high in the evaluated period, while in 220 cases the disease was not recorded in none of the years evaluated, which may reflect failures in the

sensitivity of the leptospirosis surveillance system in the state. Hotspot analysis proved to be useful for identifying clusters with high leptospirosis incidence rates in the state of Rio Grande do Sul. Some municipalities with high incidence were not identified with greater intensity on the map, which can be explained by the significant difference in the municipalities territorial extension in the south of Rio Grande do Sul, and the metropolitan macroregion of Porto Alegre being the most populated axis of the state (Rio Grande do Sul, 2020). With different approaches, other authors have already verified the same region of Rio Grande do Sul as a high-risk cluster for disease (Barcellos et al., 2003; Galan et al., 2021), which demonstrates the maintenance of incidence clusters in the state over the last decades.

In Brazil, leptospirosis is an endemic disease distributed in all regions, with a higher prevalence in the South and Southeast regions (Marteli et al., 2020; Galan et al., 2021). In urban areas, leptospirosis transmission greatest risk occurs in areas with poor sanitation infrastructure, precarious housing and prone to natural flooding caused by rains (Chaiblich et al., 2017). The metropolitan macroregion of Porto Alegre, which constitutes a cluster of cases in the last decade, concentrates 38.2% of the total population of the state (Rio Grande do Sul, 2020) is a low altitude area, where cities often experience periodic flooding, which can facilitate disease transmission in the form of outbreaks (Schneider et al., 2015).

In southern Brazil, leptospirosis incidence in rural areas is twice as high as in urban areas (Galan et al., 2021). About 50% of the municipalities in Rio Grande do Sul are considered at risk for the disease, most of which are leptospirosis critical areas (Schneider et al., 2015). The concentration of rural cases of leptospirosis in the state is reported in the central region, which produces tobacco and the south, which produces rice (Schneider et al., 2015), with a higher incidence among males (Galan et al., 2021). A higher incidence of the disease in the regions is mentioned in the literature with risk for rural workers, which alerts to the danger of infection in those places, with the presence of synanthropic animals, as well as exposure to production animals that may be infected. Even with worker health surveillance efforts, there is great difficulty in adapting workers from rural segments to accept personal protective equipment use. Thus, preventive strategies are increasingly necessary and must be intensified, through educational campaigns for this sector.

In the present study, leptospirosis seasonality was observed, with a higher incidence in the hottest and wettest months. Warnasekara et al. (2021), using SARIMA

time series analysis, observed a higher leptospirosis incidence in wetlands in Sri Lanka, which also had higher rainfall and a higher number of rainy days per month. Still, they attribute the difficulty of Leptospira to survive for long periods to low temperatures, high altitude and high solar radiation. In Rio de Janeiro, from 2007 to 2012, Guimarães et al. (2014), observed leptospirosis seasonality, with a higher concentration in the summer, and increase trend in the number of cases in the period 2008-2010, due to the rain precipitation increase. Through time series analysis, they suggested that there is a time interval between precipitation peak and the symptoms appearance, in which the number of cases begins to increase about a month after the rainy season. This information becomes crucial for preventive or assistance actions planning for the exposed communities.

The knowledge of the temporal dynamics and predicting infectious disease outbreaks using time-series, and particularly the SARIMA and GAM models, is the goal of several researchers (Martinez and Silva, 2011; Warnasekara et al., 2021). GAM models are often used in association analysis that do not show a linear pattern. This is the situation that usually occurs with the temporal evolution of infectious diseases. Thus, in this study, the GAM model includes non-linear effects of time and leptospirosis incidence allowed a better visualization of the temporal dynamics of the disease in Rio Grande do Sul, based on the identification of temporal trends and significant occurrence peak in southern Brazil, mainly in 2011, 2014 and 2019. In 2020, on the other hand, the SARIMA model predicted a fall in incidence in the first half, followed by an increase in the second half of 2020.

The estimation of the occurrence of future cases, based on predictive models such as SARIMA (1,2,1) (1,0,1), which offer an acceptable adjustment, can be highlighted as one of the tools available to face the challenges to the surveillance of leptospirosis and other infectious diseases in Brazil. The autoregression and moving average parameters showed that leptospirosis cases number in future periods can be estimated by leptospirosis cases number that occurred in the previous months. The proximity of leptospirosis real number cases that occurred in 2019 (5.9 cases / 100,000 inhabitants) and SARIMA predicted number (3.9 cases / 100,000 inhabitants; CI. 95%=1.9 – 6.7) showed that the model can be used to predict leptospirosis cases in the state. Thus, the prediction of cases as an instrument for planning control actions at the municipal level, associated with other determining variables and preventive measures,

can highlight priorities in small areas of the urban territory and indicate the adoption of integrated strategies (Martinez e Silva 2011; Gabriel et al. 2019).

Using secondary data from surveillance systems entails working with numerous limitations. We used secondary data where cases were reported by health professionals, health services, and the public. Therefore, this case reporting was subject to misclassification bias. Thus, the municipal epidemiological surveillance service must be attentive and the health services sensitive to the notification of this disease, with professionals trained in the identification of suspected cases of the disease and notification, in order to ensure early diagnosis and treatment, in addition to being active in epidemiological investigation and health education actions for the population. The limitations of the study were, among others, related with this cited misclassification bias, especially if we consider that in almost half of the state municipalities (220; 44.2%) no leptospirosis cases were reported in any of the 13 evaluated years. However, even with the possible notification biases, the analysis of these data is extremely valuable for health agencies, as it is a way to analyze the behavior of various health problems and thus direct efforts and resources in order, to anticipating risk situations, and then to make surveillance and control more effective.

This study evaluated the spatial and temporal leptospirosis dynamics in Rio Grande do Sul, and showed the highest incidence regions and the highest infection risk years, allowing a satisfactory disease incidence prediction. Through the used models, we bring an approach that intends to help the understanding of the disease occurrence in an endemic area in southern Brazil. In addition, the used prediction model can be made dynamic, after current data inclusion, as well as more complex predictive models can be developed taking into account climatic, environmental and socioeconomic variables, among others, for a more accurate prediction. The model can be applied over timeframes or at smaller geographic levels to predict leptospirosis incidence. Thus, we consider that proposed leptospirosis incidence prediction serves as a tool for surveillance services, considering the growing need to clarify the dynamics of this neglected disease in Brazil.

Author Contribuition

Conceptualization: Alessandra Jacomeli Teles, Fábio Raphael Pascoti Bruhn.

Data curation: Bianca Conrad Bohm, Fábio Raphael Pascoti Bruhn.

Formal analysis: Nádia Campos Pereira Bruhn, Bianca Conrad Bohm, Fábio Raphael Pascoti Bruhn.

Investigation: Alessandra Jacomeli Teles, Fábio Raphael Pascoti Bruhn, Bianca Conrad Bohm.

Methodology: Alessandra Jacomeli Teles, Fábio Raphael Pascoti Bruhn, Bianca Conrad Bohm, Nádia Campos Pereira Bruhn.

Project administration: Fábio Raphael Pascoti Bruhn.

Software: Fábio Raphael Pascoti Bruhn, Bianca Conrad Bohm, Nádia Campos Pereira Bruhn.

Validation: Alessandra Jacomeli Teles, Fábio Raphael Pascoti Bruhn, Bianca Conrad Bohm, Suellen Caroline Matos Silva, Nádia Campos Pereira Bruhn.

Writing – original draft: Alessandra Jacomeli Teles.

Writing – review & editing: Suellen Caroline Matos Silva.

Acknowledgements

We are also grateful to the SUS/RS - Sistema único de Saúde of Rio Grande do Sul - workers not only for the infectious diseases control and mitigation actions, but also providing fundamental data for the developed analysis.

References

Adler B, Moctezuma A. Leptospira and leptospirosis. VetMicrobiol. 2010;140(3):287-296.

Anselin L. Local Indicators of Spatial Association—LISA. In: Geografichal analysis. Volume27, Issue2, p. 93-115, 1995. https://doi.org/10.1111/j.1538-4632.1995.tb00338

Antunes, J.L.F.; Cardoso, M. R. A. Uso da análise de series temporais em estudos epidemiológicos. *Epidemiol. Serv. Saúde*, Brasília, 2015; 24(3):565-576.

Atlas Socioeconômico do Rio Grande do Sul. 6th ed. e atual. Rio Grande do Sul: [publisher unknown]; 2021. 205 p. ISBN: 978-65-87878-00-3.

Baquero OS, et al. Dengue forecasting in São Paulo city with generalized additive models, artificial neural networks and seasonal autoregressive integrated moving average models. PLoS ONE. 2018;13(4)

Barcellos C, et al. Distribuição espacial da leptospirose no Rio Grande do Sul, Brasil: recuperando a ecologia dos estudos ecológicos. Caderno de Saúde Pública. 2003;19(5):1283-1292.

Benacer D, et al. Epidemiology of human leptospirosis in Malaysia. Acta tropica. 2016; 157(1):162–168.

Buffon EAM. Vulnerabilidade socioambiental à leptospirose humana no aglomerado urbano metropolitano de Curitiba, Paraná, Brasil: proposta metodológica a partir da análise multicritério e álgebra de mapas. Saúde Soc. 2018; 27(2): 588-604.

Chaiblich JV, et al. Estudo espacial de riscos à leptospirose no município do Rio de Janeiro (RJ). Saúde Debate. 2017;41: 225-240.

Clazer M, et al. Leptospirose e seu aspecto ocupacional - revisão de literatura. Arq. Ciênc. Vet. Zool. UNIPAR. 2015; 18:191-198.

Conceição G.M.S, et al. Modelos GLM e GAM para análise da associação. Revista Brasileira de Epidemiologia. 2001;4: 206-219.

Dickey D.A, Fuller W.A. Distribution of the Estimators for Autoregressive Time Series with a Unit Root. Journal of the American Statistical Association. 1979;74: 427-431.

Galan DI, Roess AA, Pereira SVC, Schneider MC (2021) Epidemiology of human leptospirosis in urban and rural areas of Brazil, 2000–2015. PLoS ONE 16(3): e0247763. https:// doi.org/10.1371/journal.pone.0247763.

Gonçalves NV, et al. Distribuição espaço-temporal da leptospirose e fatores de risco em Belém, Pará, Brasil. Ciência e Saúde Coletiva. 2016;21(12):3947- 3955.

Guimarães RM, et al. Análise temporal da relação entre leptospirose e ocorrência de inundações por chuvas no município do Rio de Janeiro, Brasil, 2007-2012. Ciência e Saúde Coletiva. 2014;19(9):3683- 92.

Hastie T, Tibshirani R. Generalized additive models: some applications. J. Am. Stat. Assoc. 1987;82(398):371-386.

T Hastie, R Tibshirani. Generalized Additive Models. John Wiley & Sons, Inc [Internet]. 1990 [cited 2021 Jun 2]; DOI 10.1002/9781118445112.stat03141. Available from: https://onlinelibrary.wiley.com/doi/epdf/10.1002/9781118445112.stat03141.

Ho CC, Ting CY. Time Series Analysis and Forecasting of Dengue Using Open Data: Advances in Visual Informatics. Springer. 2015; 51–63. DOI:10.1007/978-3-319-25939 0_5:51–63.

IBGE. Sinopse do Censo Demográfico de 2010 [Internet]. Instituto Brasileiro de Geografia e Estatística; 2010 [Sep 18; cited 2021 Apr 23]. Available from: https://www.ibge.gov.br/apps/populacao/projecao/.

IBGE. Sinopse do Censo Demográfico de 2010 [Internet]. Instituto Brasileiro de Geografia e Estatística; 2021 [revised 2020 Sep 18; cited 2021 Apr 23]. Available from: https://www.ibge.gov.br/apps/populacao/projecao/.

Lal V, et al. Forecasting incidence of dengue in Rajasthan, using time series analyses. Indian J Public Health. 2012; 56:281-285.

Ljung G.M, Box G.E.P. On a measure of lack of fit in time series models. Biometrika. 1978;65: 29–303.

Kapagunta C, Chetty P. Structural Time Series Modelling of Crime Rates [Internet]. 2. [place unknown]: GRIN Verlag; 2013 [revised 2013 Sep 25; cited 2021 Sep 25]. 120 p. ISBN: 9783656548690. Available from: https://www.projectguru.in/time-series-and-forecasting-models-in-disease-epidemiology/.

Marteli MA, et al. Análise espacial da leptospirose no Brasil. Saúde Debate. 2020;44: 805-817.

Martinez AZ, Silva EAS. Predicting the number of cases of dengueinfection in Ribeirão Preto, São Paulo State, Brazil, using a SARIMA model. Cadernos de Saúde Pública. 2011;27(9):1809-1818.

Martins MHM, Spink MJP. A leptospirose humana como doença duplamente negligenciada no Brasil. Ciência &Saúde Coletiva, 2020; 25(3):919-928.

McCullagh, P. Generalized linear models. Eur. J. Oper. Res., 16 (3) (1984): 285-292.

Ministério da Saúde. Define a Lista Nacional de Notificação Compulsória de doenças, agravos e eventos de saúde pública nos serviços de saúde públicos e privados em todo o território nacional, nos termos do anexo, e dá outras providências. Diário Oficial Da União [Internet]. 2016 Feb 17 [cited 2016 Feb 17]; 1:23. Available from: https://saude.rs.gov.br/upload/arquivos/201703/21120656-5-1-portaria-204.pdf.

Morettin PA, Toloi CMC. Análise de Séries Temporais. 1th ed. São Paulo: Edgard Blucher; 2006. 544 p. ISBN: 978-8521203896.

Promprou S, Jaroensutasinee M, Jaroensutasinee K. Forecasting dengue haemorrhagic fever cases in southern thailand using ARIMA models. Dengue Bulletin [Internet]. 2006 [cited 2021 May 19];30 DOI 0250-8362. Available from: https://apps.who.int/iris/handle/10665/170355

Qgis. QGIS Geographic Information System: QGIS Association. [Internet]. [place unknown]; 2021 [cited 2021 Jul 19]. Available from: http://www.qgis.org.

Ravindra K, et al. Generalized additive models: Building evidence of air pollution, climate change and human health. Environment International. 2019;132

Secretaria de Vigilância em Saúde. Guia de Vigilância em Saúde [Internet]. 3rd ed. Brasília: [publisher unknown]; 2019 [cited 2021 Jun 16]. 741 p. único vol. ISBN: 978-

85-334-2706-8. Available from: https://bvsms.saude.gov.br/bvs/publicacoes/guia_vigilancia_saude_3ed.pdf

Secretaria de Vigilância em Saúde [Internet]. Leptospirose: Situação epidemiológica do Brasil no período de 2007 a 2016. 1996- Brasília: [publisher unknown]; Vol. 49, No. 41, 2018 [cited 2021 May 16]. ISSN: 9352-7864. Available from:https://www.researchgate.net/publication/328171612_Leptospirose_situacao_epidemiologica_do_Brasil_no_periodo_de_2007_a_2016.

Schneider MC, et al. Leptospirosis in Rio Grande do Sul, Brazil: An Ecosystem Approach in the Animal-Human Interface. PLoS Negl Trop Dis. 2015;9 (11)

Silva FJ, et al. The importance of Leptospira interrogans serovars Ictero haemor rhagiae and Canicola in coastal zone and in Southern fields of Rio Grande do Sul, Brazil. PesqVetBras. 2014;34(1):34-38.

Souza, VMM, et al. Avaliação do sistema nacional de vigilância epidemiológica da leptospirose – Brasil, 2007. Cadernos Saúde Coletiva, 2010; 18 (1):95-105.

Souza IPO, Uberti MS, Tassinari WS. Geoprocessing and spatial analysis for identifyingleptospirosis risk areas: a systematic review. Revista Instituto de Medicina Tropical São Paulo. 2020;62:e35.

Warnasekara, J; Agampodi, S.; Abeynayake RR. Time series models for prediction ofleptospirosis in different climate zones in Sri Lanka. PLoS ONE, 2021. 16(5): e0248032.

World Health Organization. Human leptospirosis: guidance for diagnosis, surveillance, and control. Geneva, World Health Organization; International Leptospirosis Society, 2003. 109p.

Zhang X, Zhang T, Young AA, Li X. Applications and Comparisons of Four Time Series Models in Epidemiological Surveillance Data. PLoS ONE 2014; 9: e88075.
