# Supplementary material for: Spatial and temporal dynamics of leptospirosis in South Brazil: A forecasting and nonlinear regression analysis
Source: PLoS Negl Trop Dis. 2023 Apr 14;17(4):e0011239. doi: 10.1371/journal.pntd.0011239 (PMC10132658; doi:10.1371/journal.pntd.0011239)
Supplement: Attachment — Submitted filename: Rebuttal Letter 03012023 4.docx.pdf [file pntd.0011239.s006.pdf]

Rebuttal Letter

Pelotas, january 04, 2023.

PNTD-D-22-01154

Dear,

You will find the point-by-point response to the suggestions requested by the reviewers. We appreciate the kindness and availability of these reviewers, and the modifications along the text will undoubtedly contribute to improving our manuscript's quality.

We hope you have interest to publish our manuscript.

Sincerely, on behalf of all authors,

Fábio Raphael Pascoti Bruhn, Ph.D.
Associate Professor; Federal University of Pelotas, Brazil.

COMMENTS ON MANUSCRIPT

2 Specific comments

2.1 Major issues

- Authors should improve explanations about the implementation of the models. Include additional results (as a supplementary material or within the manuscript) that supports the selection process of the model.
–The authors mentioned that the models were trained, but no further discussion is provided

Answer:

Seeking to respond to the reviewer's request, that the authors should improve explanations about the implementation of the models, we have changed the GAM methodology and added additional results as suplementary information that support the selection process of the model (S1 Table and S1 Fig):the tables with the parameters used in the selection process of the GAM distributions and terms, as well as the graphs constructed from the different models.

- It is not sufficient to say that “we followed Baquero et al. (2018) and used a Poisson likelihood and cubic splines with 5 knots on all predictors ”. The authors need to explain the rationale of why this Poisson-GAM is adopted for the presented work
– Negative binomial and Gaussian likelihoods were also used in the training process, but no comments are provided about these comparisons

Answer:

In GAM analysis we tested the families of poissson, gaussian and negative binomial distributions, and all results (parameters for selection of models and smoothing terms) are found, in this new version, in S1 Table and S1 Fig. However, considering that the response variable is a count (Leptospirosis cases confirmed in year, offset term the population) and following Wood (2017) and Cota et al (2021), the Poisson distribution is the reasonable distribution to use. We were aware of the fact that the selection methods are only appropriate for selecting between reasonable models. Following the reviewer's recommendation, we present the results of the other tested families (Gaussian and negative binomial) as supplementary information in the article.

- While the authors provide plots of the results, they omitted vital information on how the models were ultimately chosen
–No predictors were explicitly mentioned
–Results on predictive performances and model comparison (AIC) need more explanation (see first bullet provided)
- Explanations in Methods and Results sections need to be improved/restructured

Answer:

Specifically on the parameters of the forecasting models (SARIMA), new references were added, which justify the use of selection criteria for the models used in the research (AIC for choosing among the models; Mean Absolute Percent Error and Theil-U statistic for evaluating the prediction). Citations are added below, with methodological details on the used techniques: Burnham, 2002; Borrego-Morell et al., 2021; Ma et al., 2021.

Furthermore, as for the GAM models, a table with the parameters of each tested SARIMA model has been added (S2 Table).

-When explaining the prediction component, the intervals provided should be presented as prediction intervals, not as confidence intervals

Answer:

We agree with this review. The word has been corrected to "prediction intervals".

-In Figure 4, use prediction bands instead of lines, and use different color (not light green)

Answer:

Thanks for the suggestion, we have modified the colors to improve the image visualization.

-I suggest the authors to get editing help from someone with full professional proficiency in English

Answer:

We have sent the corrected article for English revision by a certified company. We hope you agree.

-The references of the manuscript should follow the journal's guidelines. It is somewhat difficult to read through the manuscript when several citations are located within a sentence given the style provided

PLOS Guideline: “References are listed at the end of the manuscript and numbered in the order that they appear in the text. In the text, cite the reference number in square brackets (e.g., “We used the techniques developed by our colleagues [19] to analyze the data”). PLOS uses the numbered citation (citation-sequence) method and first six authors, et al.”

Answer:

We apologize for this mistake, the references have been updated in accordance with the journal's guidelines.

2.2 Minor issues

-Abbreviations in the Abstract should be avoided In the sentence “ Leptospirosis behavior over the years was evaluated by time series analyses, using generalized additive models (GAM) and SARIMA models to predict its future incidence.”

-in the Abstract, remove the two abbreviations. Provide the full names of the methods in the Abstract

Answer:

Thanks for the suggestion. We updated the words, as requested in the abstract.

-Provide the full model name for SARIMA when first introduced in the text
Example: Seasonal Autoregressive Integrated Moving Average (SARIMA)

Answer:

Thanks for the suggestion. We updated the words, as requested.

-Check several words not in English (Portuguese): endˆemic, ´areas

Answer:

We are sorry that this mistake was not detected before submission, it has now been corrected.

-Change keyword from “surviellence” to “surveillance”

Answer:

The word has been corrected to “surveillance”.

-Remove sentence “Leptospirosis is a persistent health issue in Brazilian south” from Abstract since the first sentence states a similar idea

Answer:

We agree with this review and removed the sentence from Abstract.

-Provide meaning for “sp.” as specie(s) and then use Leptospira sp.

Answer:

Thanks for the suggestion, we inserted in the introduction as requested.

"Leptospirosis is caused by Leptospira interrogans, a bacterial species that survives in varied environments for prolonged periods and affects humans and wild, domestic, and synanthropic animals, which then become carriers of this species and contribute to its spread in nature"

-Provide an Author Summary section, in which the research findings are accessible to a wide audience. See journal guidelines for this component

Answer:

We are sorry that this mistake was not detected before submission. Author Summary have been inserted.

-Abbreviations are being defined in multiple occasions. Define them once (only the first time they are mentioned)

Answer:

Thanks for the suggestion, and we changed it accordingly.

-Do not start a new paragraph with "Thus,..."

Answer:

Thanks for the suggestion, the alterations were done.

-There are several typos within the text . Example of typos: colected, clursters, redictor

Answer:

We are sorry that this mistake was not detected before submission and in the English review, it has now been corrected.

-Remove "(clursters)" in the sentence " This analysis was performed to detect spatial clusters with a higher concentration of high incidences (clursters) of leptospirosis"

Answer:

The word was deleted and we changed it accordingly.

-Final versions of the Figures should be in high quality resolution (use TIFF). It is not possible to Zoom-in very well in the figures

Answer:

The figures has been re-done and have been changed to tiff.

-Revise sentence "GAM models provide a framework for generalizing a general linear model,allowing the variables nonlinear functions additivity ". The phrase "generalizing a general linear model" is not providing the correct idea

***Answer**:*

Following this and other suggestions from the reviewer, the GAM methodology part was redone, including changes to this paragraph.

-Improve explanation in second paragraph of subsection Generalized additive models (GAM). , within the explanation? This paragraph is an example of a text that can be either removed from the text or improved

Answer:

Following this and other suggestions by the reviewer, the GAM methodology part was redone, including the removal of this paragraph.

-Provide citation of the R packages: "mgcv" and "tidyverse"

Answer:

Right, the citation have been inserted in the text.

-In "(60 deaths, LR 8.9%)", please provide the meaning of LR. Is it lethality rate? Please, define the abbreviation before using the abbreviation

Answer:

We agree and this abbreviation was deleted. We have kept only "incidence rate" and "lethality rate" throughout the text.

-When referencing a figure or table, use expression "Figure 1" or "Table 1"

Answer:

Right, the expression was updated.

-What happened with the data from 2007 to 2009 in the incidence and lethality maps, and heatmaps? It is not clear from the text.

Answer:

In fact, the time frame used in the analyzes was not standardized. Thanks for the insight into this. In the new version, all analyzes were redone using the correct cutout, from 2007 to 2019.

-Do not use the phrase "GAM models ". The M in GAM means model

Answer:

Right, we removed the sentence.

-Provide references for all softwares. I do not see references for R and Gretl softwares

Answer:

Right, the references have been inserted.

-The following paragraph should be included as an Ethic Statement: " The study was approved by the Ethics Committee of the Faculdade de Medicina of Universidade Federal de Pelotas, CAAE 46714421.0.0000.5317, in accordance with all ethical principles and current legislation for research involving human beings. In this way, data confidentiality was guaranteed and used only for research purposes.'

Answer:

We have included the paragraph as an Ethic Statement

-In Discussion section, address what are possible future works (e.g. further analysis into under-reporting and miss clasification)

Answer:

We agree with the reviewer. A discussion of possible future studies has been added to the last paragraph of the discussion.

"Future studies could use the results obtained through these models to evaluate other time frames or smaller geographic areas, particularly those in which the disease is more frequent, for example, the Centro Oriental Rio Grandense and the Metropolitan of Porto Alegre mesoregions. It would be important to conduct studies that assess the causality of leptospirosis in these regions with the highest occurrence, especially considering its occurrence profile, which is more linked to the urban or rural environment. This could also address the high underreporting observed in most municipalities, especially those located in the southern and western regions of Rio Grande do Sul."

EMAIL

- Objective requires a minor revision related to the resolution for spatial analysis and time-series and forecasting analysis.

Answer:

Following this and other suggestions from the reviewer, the objective section has been changed.

- Figures quality requires revision.

Answer:

Following this and other suggestions, the figures were changed, including changing the format to TIFF.

- Adequate to the objective, even though time series analysis could be improved.

Answer:

Following this and other suggestions by the reviewer, the methodology and presentation of the results of the time series analyzes were changed (GAM and SARIMA), including the addition of more results in the article supplementary material.

- I suggest applying GAM and SARIMA to analyse the "cluster municipalities" temporal behaviour of leptospirosis incidence.
- To obtain a stronger evidence, municipalities with enough data (Porto Alegre and couple more) could be analysed using GAM and SARIMA.
- GAM and SARIMA over selected municipalities could contribute to the analysis.

Answer:

Thanks for the reviewer's suggestion. In this new version, we inserted specific descriptive analyzes for the clusters of cases observed between 2007 and 2019, present in the Centro Oriental Rio Grandense and Metropolitana de Porto Alegre mesoregions. In these

analyses, indicators of morbidity, lethality and mortality from leptospirosis were included, which was very useful to demonstrate the relevance of this part of the state in the occurrence of leptospirosis. These results can help public health services in allocating efforts and resources to control the disease, specifically in these regions. This is especially important in Brazil, where resources for public health, including surveillance, are scarce. These analyzes also demonstrated that the lack of cases in other regions demonstrates underreporting in the state, which is also a warning to surveillance services.

I would like to clarify that this article is the result of a line of research on leptospirosis in Rio Grande do Sul. This is an exploratory work, initially developed using data from the entire state. As reported in the final considerations of this article, there is a lack of knowledge about the epidemiology of leptospirosis in the state. Thus, epidemiological studies, especially those developed in smaller geographic units, can help in a deeper understanding of the causes of this high number of cases in the state, mainly in these two mesoregions observed as clusters in the present research.

We understand, as suggested by the reviewer, that further analysis of these areas with more cases, using GAM and SARIMA, could provide relevant information for understanding the disease in the state. However, this is already being developed in these areas by the team linked to this line of research, including the use of these same methodologies, such as the GAM, in the elucidation of the causes of leptospirosis in these regions, mainly considering the different profiles of the disease in these mesoregions (rural and urban).

Thus, understanding that the present work: i. it is an exploratory survey of the entire state, which raises hypotheses to be tested in other studies; i. presents statistical analyzes and modeling never previously applied to data on leptospirosis in the region, and iii. that there is still a lack of information about this disease in the region, mainly due to the fact that it is a neglected and underreported disease, despite its high morbidity and lethality, we chose not to present in this article the results of the application of these spatial statistical modeling methodologies and temporal specify the regions with the highest occurrence of the disease. We believe that the epidemiological analysis of these mesoregions, especially considering the relevance of the disease, should be carried out in depth, including with a focus on understanding the chain of causality of leptospirosis in the region.

We are very grateful for this suggestion, as it reinforces the same perception of the team about the analysis of data in this Brazilian state. In addition, we are available to reflect and discuss with the editors and reviewers in this regard, so that we can develop an article with an impact on public health in a region as lacking in epidemiological information as Rio Grande do Sul.

Article Text

- ii) clearly state that spatial analysis is at municipality level and that time-series analysis and prediction is at state resolution to avoid misunderstandings.

Answer:

We agree with the reviewer. We changed the objectives to meet the suggestion. In addition, we added to the methodology this difference in the levels of analysis used in the spatial and temporal models.

"*The present study aimed to analyze the spatial and temporal dynamics of leptospirosis incidence and lethality rates during 2007–2019 at the municipal and state levels in Rio Grande do Sul, Brazil, and to predict the expected incidence of the disease in the subsequent period."*

"These indicators were annually mapped in all municipalities in Rio Grande do Sul to confirm the existence of clusters and thus analyze the disease risk in different regions of the state. The dynamics of the incidence rates at the state level between 2007 and 2019 were analyzed by a generalized additive model (GAM), while the number of cases for 2020 for Rio Grande do Sul was forecasted using a seasonal autoregressive integrated moving average (SARIMA) model."

-the last two years? or just 2019?

Answer:

By GAM, it is possible to observe an increase in risk from 2018, therefore, in the last 2 years evaluated there is an increase. However, in order not to create confusion with the results of the descriptive time series, we have changed the text, as follows:

"The studied time-series highlighted the difficulties in controlling this disease, given the increase in the number of leptospirosis cases in the last years of the evaluated time series."

-In urban areas it seems clear the "dynamics". Make clear in the next paragraph the "dynamics" in rural municipalities.

Answer:

This discussion was really missing. In order to respond to this reviewer's suggestion, we have changed the first five paragraphs of the discussion. We included information on the relevance of leptospirosis in Rio Grande do Sul and the dynamics of incidence in the two main clusters of the disease observed in the state, one of them predominantly rural economic activity (Centro Oriental Rio Grandense mesoregion), and a more urban one (Mesoregion Metropolitana de Porto Alegre).

"Nevertheless, in 2019, the incidence rate of leptospirosis in Brazil was 1.73 cases/100,000 inhabitants, while in Rio Grande do Sul, it was almost four times higher, reaching 6.1 cases/100,000 inhabitants [45]. Hotspot analysis proved to be useful for identifying clusters of leptospirosis, as this analysis identified clusters located in the mesoregions with the highest incidence rates in the state: Metropolitan of Porto Alegre (4.08 cases/100,000 inhabitants) and Centro Oriental Rio Grandense (16.7 cases/100,000 inhabitants). The municipalities in these mesoregions have the highest population density, and a smaller territorial extension in Rio Grande do Sul [46]. The Metropolitan of Porto Alegre is the most urbanized mesoregion (93.2%), while the Centro Oriental Rio Grandense is the least urbanized mesoregion (69.8%) in the state [46]. These characteristics indicate that these mesoregions have different profiles of disease occurrence, predominantly urban or rural, and constitute the two highest risk areas for human leptospirosis in the state in all years between 2007 and 2019. By using different approaches, other authors have already confirmed that the same regions of Rio Grande do Sul are high-risk clusters for the disease [10,47]; this finding demonstrates the maintenance of incidence clusters in the state over the last decades."

-Include some discussion about mortality/lethality

Answer:

We inserted the following paragraph in the discussion, with lethality and mortality data, with the aim of demonstrating the relevance of the disease in Rio Grande do Sul, and the existence of a health problem, especially in the mesoregions identified as clusters of the disease in the state between 2007 and 2019.

"With regard to deaths due to the disease, between 2007 and 2019, the average leptospirosis lethality was 5.3% in Rio Grande do Sul, while it was 8.6% (56.6% higher) in the Metropolitan of Porto Alegre mesoregion. The lethality rate was lower at 4.0% in the Centro Oriental Rio Grandense mesoregion. On the other hand, this mesoregion had the highest mortality rates due to leptospirosis from 2007 to 2019, with an average of 0.61 deaths/100,000 inhabitants, which was higher than the average rates for the entire country (0.17 deaths/100,000 inhabitants) [45], Rio Grande do Sul (0.23 deaths/100,000 inhabitants), and the Metropolitan of Porto Alegre mesoregion (0.32 deaths/100,000 inhabitants). These high morbidity and mortality indicators demonstrate the severity of leptospirosis in Rio Grande do Sul and reinforce the importance of allocating efforts to control this disease in the state, particularly in the regions of the clusters identified in the present study in all years between 2007 and 2019."

-As this is not of general knowledge, a brief explanation in Methods or an additional plot of temperature and precipitation in results could be useful.

Answer:

Following the reviewer's suggestion, to clarify the objective of the seasonality analysis in the context of Rio Grande do Sul, we added a sentence to the methodology, specifically in the place that addressed the seasonality analysis.

"Rio Grande do Sul experiences hotter months from November to April and colder months from May to October [19,34]. In the present study, seasonality was tested to detect whether leptospirosis occurrence was concentrated in some of these months."

-Discuss the importance of GAM showing these peaks. If not it seems that SARIMA was good enough for the analysis.

Answer:

In order to meet the reviewer's suggestion, the following paragraphs were added to the methodology and discussion, with the aim of making clear the differences in the objectives of the two used techniques: GAM, used to evaluate the temporal dynamics, and SARIMA, used in the process forecast cases for the future.

"These indicators were annually mapped in all municipalities in Rio Grande do Sul to confirm the existence of clusters and thus analyze the disease risk in different regions of the state. The dynamics of the incidence rates at the state level between 2007 and 2019 were analyzed by a generalized additive model (GAM), while the number of cases for 2020 for Rio Grande do Sul was forecasted using a seasonal autoregressive integrated moving average (SARIMA) model."

"In GAM, which was used to evaluate the temporal dynamics between 2007 and 2019, a smoothing function was used in the year variable to confirm the dependence of observations on time to recognize the functional trend of the "year" variable [30]. Thus, it

was possible to observe growth trends in the incidence rate, mainly from 2016 to 2019, in addition to peaks noted in the years 2010, 2014, and 2019. The SARIMA model used to predict cases enabled us to observe a perspective of an increase in cases, especially in the second half of 2020."
