# Supplementary material for: Spatial and temporal dynamics of leptospirosis in South Brazil: A forecasting and nonlinear regression analysis
Source: PLoS Negl Trop Dis. 2023 Apr 14;17(4):e0011239. doi: 10.1371/journal.pntd.0011239 (PMC10132658; doi:10.1371/journal.pntd.0011239)
Supplement: Attachment — Submitted filename: Comments on Manuscript PNTD-D-22-01154_R1.pdf [file pntd.0011239.s007.pdf]

Resubmission: Comments for Manuscript PNTD-D-22-01154-R1

1 Summary

Manuscript title: Spatial and temporal dynamics of leptospirosis in South Brazil: a forecasting and nonlinear regression analyses

Main research question/contribution: The manuscript focuses on the implementation of Generalized Additive Models (GAM) and Seasonal Autoregressive Integrated Moving Average (SARIMA) models to forecast leptospirosis incidence cases and to identify regions (clusters) with high leptospirosis incidence and lethality rates in the state of Rio Grande do Sul.

Revised - Overall impression: I appreciate the detailed point-by-point response of each comment from authors' original submission. After careful reading of the revised manuscript, the authors has improved the quality and readability of the manuscript by following reviewers' suggestions. The manuscript requires very minimal "minor" modifications. Please refer to the Specific Comments section for more details.

Reviewer decision on the revised version: Based on the revised form of the manuscript and its improved quality, I recommend this paper for publication, only after addressing the minor issues described below. Therefore, my decision is "**Minor Revision**".

2 Specific comments

2.1 Major issues

- No additional major issues are present in this version. All of the major issues from original review were carefully addressed, with exception of quality of figures. **Refer the last bullet point in minor issues

2.2 Minor issues

- Typo: A space in "leptospirosisis" is needed to say "leptospirosis is". This is located in first sentence of the Abstract. In specific, line 16
- In line 179, the index "i" for year is not written as a mathematical formula
- In line 184, the term "GAM model" needs to be change to just "GAM" since the M in GAM means model
- The label for the 95% prediction interval in Figure 5 is still denoted as IC. I assume it is for "intervalo de confiança". Authors could use "P-I" or "PrI" for labeling the prediction interval and make sure it is clearly defined in the caption

- **I recommend the authors to search for an alternate format for the Figures within the QGIS and R softwares. Even after modifying the format to TIFF, the figures are still pixelated when you zoom-in, which makes it hard for readers to carefully explore each map or plot individually. When looking at the full sized figures, no reading issues are noted. A good thing is that the revised text is very informative on what the plots represent. I suggest exporting figures using resolutions of 600 DPI (dots per inch) for figures in black and white and 300 DPI for half tones/colour figures.
