# Supplementary material for: Spatial and temporal dynamics of leptospirosis in South Brazil: A forecasting and nonlinear regression analysis
Source: PLoS Negl Trop Dis. 2023 Apr 14;17(4):e0011239. doi: 10.1371/journal.pntd.0011239 (PMC10132658; doi:10.1371/journal.pntd.0011239)
Supplement: Attachment — Submitted filename: Rebutal letter RESUBMISSION.pdf [file pntd.0011239.s008.pdf]

Rebuttal Letter

Pelotas, february 25, 2023.

RESUBMISSION: PNTD-D-22-01154

Dear,

We are glad that the changes made have met most of the suggestions from the reviewers. In this new letter, you will find the point-by-point response to the suggestions requested by the reviewers in this new avaliation. We appreciate the kindness and availability of these reviewers, and the modifications along the text will undoubtedly contribute to improving our manuscript's quality.

We hope you have interest to publish our manuscript.

Sincerely, on behalf of all authors,

Fábio Raphael Pascoti Bruhn, Ph.D.
Associate Professor; Federal University of Pelotas, Brazil.

RESUBMISSION: COMMENTS ON MANUSCRIPT PNTD-D-22-01154

2.1 Major issues

- No additional major issues are present in this version. All of the major issues from original review were carefully addressed, with exception of quality of figures.

Answer:

We are happy that the reviewers are satisfied with most of the changes proposed in the first revision of the manuscript.

About the figures, indeed, the quality of some figures was really not suitable for publication in the journal. Some of them, when joined or altered in some way, lost their quality. So, we redid the figures, now with more dpi, as suggested. We also passed the figures through the Preflight Analysis and Conversion Engine (PACE) tool, which helped a lot in this process of improvement and adaptation to PLOS standards.

The individual figures have great resolution, but sometimes in the process of merging or altering them for placement in the manuscript, the resolution deteriorates. So, even with all the changes, if any figure is still not adequate, we are available to change and improve it again.

2.2 Minor issues

- Typo: A space in “leptospirosisis” is needed to say “leptospirosis is”. This is located in first sentence of the Abstract. In specific, line 16.

Answer:

Right, this error has been corrected.

- In line 179, the index "i" for year is not written as a mathematical formula.

Answer:

Right, this error has been corrected.

- In line 184, the term "GAM model" needs to be change to just "GAM" since the M in GAM means model.

Answer:

Right, this error has been corrected.

- The label for the 95% prediction interval in Figure 5 is still denoted as IC. I assume it is for "intervalo de confiança". Authors could use "P-I" or "PrI" for labeling the prediction interval and make sure it is clearly defined in the caption.

Answer:

We are sorry that this mistake was not detected before submission. We have added the suggested label, with proper explanation, in the figure caption.

- I recommend the authors to search for an alternate format for the Figures within the QGIS and R softwares. Even after modifying the format to TIFF, the figures are still pixelated when you zoom-in, which makes it hard for readers to carefully explore each map or plot individually. When looking at the full sized figures, no reading issues are noted. A good thing is that the revised text is very informative on what the plots represent. I suggest exporting figures using resolutions of 600 DPI (dots per inch) for figures in black and white and 300 DPI for half tones/colour figures.

Answer:

As explained above, the figures were changed, including using the Preflight Analysis and Conversion Engine (PACE) tool. Despite this, if any further changes are necessary, we are at your disposal.
